# Psychometric properties of the WHO-VFQ-20 questionnaire for assessing vision-related quality of life in Brazilian urban populations with different vision status

**DOI:** 10.1038/s41598-026-40824-9

**Published:** 2026-03-03

**Authors:** Nívea Nunes Ferraz, Adriana Berezovsky, Leon B. Ellwein, Gopal Prasad Pokharel, Rubens Belfort Jr., Solange Rios Salomão

**Affiliations:** 1 Department of Ophthalmology and Visual Science, Escola Paulista de Medicina, Universidade Federal de São Paulo, São Paulo, SP Brazil; 2 National Eye Institute, Bethesda, MD USA; 3 Nepal Eye Hospital, Kathmandu, BA Nepal; 4 Department of Ophthalmology and Visual Science, Escola Paulista de Medicina, Universidade Federal de São Paulo, Rua Botucatu 806, São Paulo, SP 04023-062 Brazil

**Keywords:** Blindness, Visual impairment, Visual functioning, Quality of life, Diseases, Health care, Medical research, Psychology, Psychology

## Abstract

This study aimed to analyze the psychometric properties of the 20-item WHO-Visual Functioning Questionnaire (WHO-VFQ-20). Ordinal data and Rasch analysis were performed to determine instrument precision (internal consistency and reliability) and validity (fit statistics and dimensionality). The WHO-VFQ-20 was administered to 606 adult participants with different ocular conditions and visual acuity levels, stratified by age, sex, and socioeconomic status, from three Brazilian urban populations—a public hospital and two non-governmental institutions located in the outskirts of Sao Paulo city. Ordinal data analysis showed a mean global score of 59.6 ± 25.0 s.d., which consistently reduced with decreasing visual acuity. Higher scores were significantly associated with better overall health, higher education, and being married/cohabitating. Cronbach’s alpha coefficient of 0.94 indicated excellent internal consistency. Rasch analysis demonstrated good measurement precision with reliability coefficients of 0.99 and 0.85 for items and persons, respectively. Three items related to ocular pain and mental well-being were identified as misfitting. The variance explained by the measures suggested multidimensionality. Analyzing the instrument as two components (visual functioning and psychosocial functioning) yielded improvement on model fit, indicating acceptable precision and validity of the WHO-VFQ-20 for assessing vision-related quality of life in adults from urban areas of a middle-income country.

## Introduction

In ophthalmological clinical practice, psychophysical measurements of vision, such as visual acuity and visual field, indicate visual system capacity and define visual impairment and blindness conditions^1,2^. However, patients’ own perspectives on their vision can also offer valuable information about their visual ability, satisfaction with vision, and how it affects their everyday lives. Over the last three decades, researchers have developed questionnaires to better understand the vision-related quality of life^3–13^ and the impact of visual disabilities caused by eye diseases and ocular treatments^14^.

The 25-item National Eye Institute Visual Function Questionnaire (NEI-VFQ-25) is one of the most used instruments in the literature^15–17^ to evaluate the quality of life and vision function. This and other similar tools were initially developed and tested in high-income countries^18,19^. Differently, the 33-item Indian Vision Functioning Questionnaire (IND-VFQ-33)^19,20^ was specifically designed to consider the implications of visual impairment for both urban and rural low-income populations in India. Considering the target individuals’ literacy levels and reading difficulties, it was developed as an interview-administered questionnaire.

The functional and psychosocial impacts of visual impairment are similar across different populations^19^. However, the cultural, behavioral, lifestyle, and geographic characteristics are essential for selecting the proper instrument for the target population^9,20,21^. For instance, the difficulties faced by people with poor eyesight in high-income countries—as going to the cinema or driving in city traffic (encompassed in the NEI-VFQ-25)—do not apply to low-income populations living in rural areas along riverbanks or in the countryside with dirt roads^19^.

In 2003, experts from the Prevention of Blindness and Deafness Division held a World Health Organization (WHO) “Consultation on Development of Standards for Characterization of Vision Loss and Visual Functioning”. After reviewing several questionnaires, including the NEI-VFQ-25 and the IND-VFQ-33^9,19,20,22^, the group proposed a reduced instrument for evaluating visual functioning and quality of life, with 20 questions on topics of general vision, ocular pain/discomfort, distance vision, near vision, glare, light/dark adaptation, color vision, functional limitations, social functioning, mental well-being, and dependence. The consultants recommended field-testing this 20-Item WHO Visual Functioning Questionnaire (WHO-VFQ-20) to validate its use through multivariate analysis of questionnaire responses in association with age, sex, socioeconomic status, and visual acuity^22^.

The purpose of this study was to analyze the psychometric properties of the WHO-VFQ-20 in assessing self-reported vision-related quality of life in adult patients across the full range of visual acuity levels, stratified by age, sex, and socioeconomic status.

## Methods

### Participants

Participants were recruited from three distinct institutions: a) the Ophthalmology Outpatient Clinic of the Federal University of Sao Paulo (Unifesp), Sao Paulo, SP, Brazil; b) the Fundação Dorina Nowill para Cegos, a non-governmental institution (NGO) for the blind in Sao Paulo city and c) the Lar das Moças Cegas, a NGO for education and rehabilitation of the visually impaired in the city of Santos located 45 miles south of Sao Paulo. Eligibility criteria included adults aged 18 years or older who could respond to the visual acuity test, complete the questionnaire, and provide informed consent.

These three recruitment sites were used to assemble a heterogeneous sample, primarily stratified by visual acuity and fully representative of the spectrum of vision impairment (including blindness), providing a multidimensional characterization of visual functioning and the implications of visual impairment/blindness for quality of life in daily activities. Therefore, in addition to the main recruitment site at Unifesp, two partner institutions for visual rehabilitation of the blind/visually impaired hosted the data collection.

This study adhered to the tenets of the Declaration of Helsinki, and written informed consent was obtained from all participants. The Committee on Ethics in Research of the Federal University of Sao Paulo (CEP/Unifesp n: 0441/2022) approved the implementation of the survey protocol. This study was part of a research protocol aimed at evaluating eye care services and the burden of visual impairment/blindness in Brazil, implemented by Unifesp with partial support from WHO. Human subject research approval of the original protocol was cleared by the WHO Secretariat Committee on Research Involving Human Subjects.

### Procedures

#### Demographics

Individuals were interviewed to obtain demographic information, including age, sex, marital status (never married/widowed, previously married, married/cohabiting), education (less than primary, primary, secondary, high school, college/university), and monthly household income in minimum wages (MW), in addition to their own perception of their general health status.

Participants were stratified by presenting binocular distance visual acuity (PBDVA > 20/25; 20/32–20/63; 20/80–20/160; 20/200–20/400; 20/500–20/1000; < 20/1000), sex, age (25–39; 40–49; 50–59; 60–69; 70 + years) and socioeconomic status based on monthly household income (≤ 1; > 1–2; > 2–4; > 4–8; > 8–16; > 16 MW). For the purposes of the study, PBDVA was measured by an experienced ophthalmic technologist for distance (4 m), using a logMAR tumbling E chart. The ophthalmological diagnosis was obtained from the participant’s medical chart and classified into cataract, glaucoma, diabetic retinopathy, age-related macular degeneration, uncorrected refractive error, and others.

#### The WHO-VFQ-20 questionnaire

**Fig. 1:**
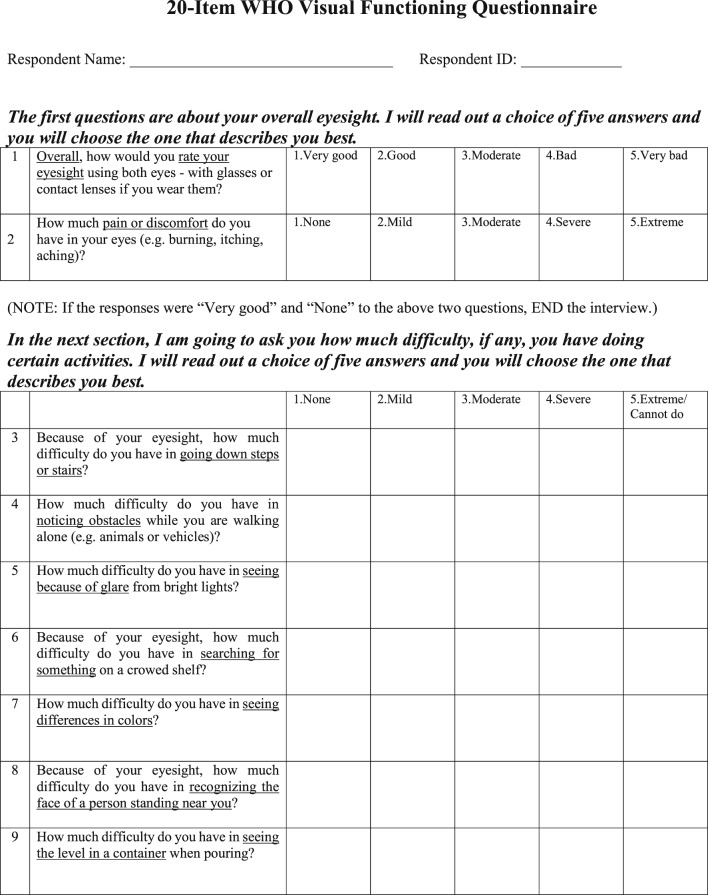

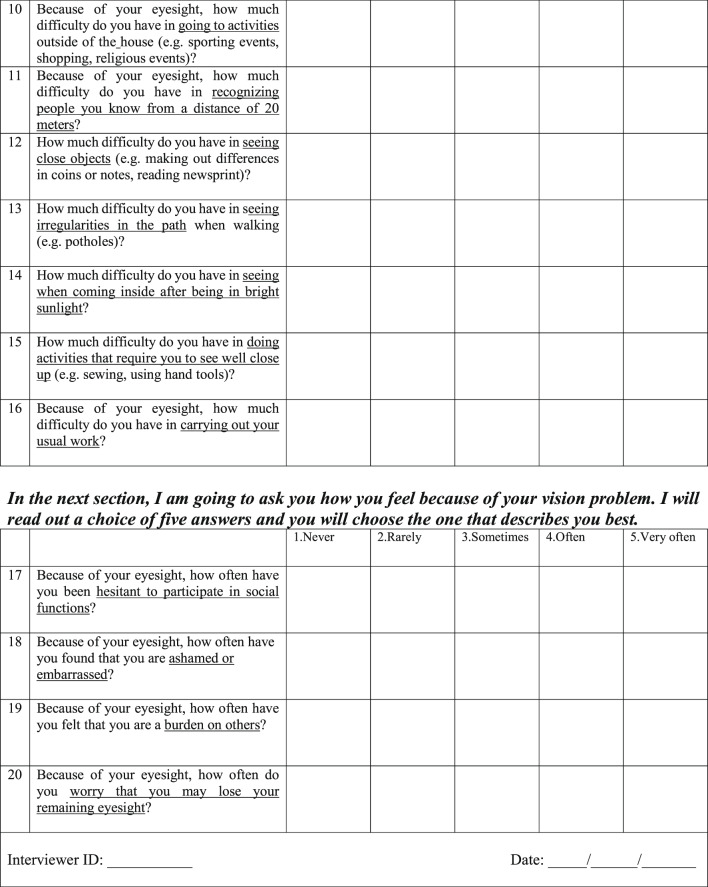
The 20-item WHO Visual Functioning Questionnaire (WHO-VFQ-20)^22^.

The WHO-VFQ-20 (presented in full in Fig. 1) was dismember in two domains: visual functioning (VF) and quality of life (QOL). The VF domain encompassed difficulties in daily activities, containing four subscales: distance vision (questions 3, 4, 11, and 13); near vision (questions 8, 9, 12, and 15); sensory adaptation, including glare disability, visual search, color vision, light–dark adaptation (questions 5, 6, 7, and 14). The QOL domain encompassed visual symptoms (ocular pain/discomfort, question 2) and psychosocial aspects, which were addressed in two subscales: social interaction, including attending social functions and working activities (questions 10, 16, and 17); mental well-being and dependency (questions 18, 19, and 20). The instrument also included a question about general vision (question 1). All questions are rated on a 5-point Likert scale, with psychometric responses ranging from “none/never/very good” (1) to “extreme/very often/very bad” (5). This scale indicates the individual’s perception when facing daily situations involving vision.

The method used for translation and adaptation of the WHO-VFQ-20 for Brazilian Portuguese was structured as follows: definition of the target audience and objectives; creation of a translation team (LBE, GPP, and SRS, a sworn Portuguese–English translator expert in ophthalmological research); forward and backward translation; comparison of the original and back-translated versions; reconciliation of differences; pretest and evaluation and final review and proofreading. The instrument was piloted to identify potential misunderstandings and to conduct cross-cultural adaptations prior to its administration in individual interviews. The pilot was conducted with 20 participants recruited from the Ophthalmology Outpatient Clinic at Unifesp, on a dedicated day set aside for interviews. Pilot data were analyzed by the translation team before proceeding with the full study and were not integrated into the study database. The final Portuguese version of the questionnaire was administered by personal interview to all participants. Careful monitoring of the data collection process avoided any missing data.

### Statistical analysis

#### Ordinal data analysis

The 5-point Likert scale was transformed into a 0 to 100 (0 representing the worst condition/maximum problem level, and 100 the best/no problem at all) score at 25-point intervals. The arithmetic mean of the domains scores were calculated as well as the global score of vision-related quality of life (combining VF and QOL domains).

WHO-VFQ-20 reliability was provided by examining the internal consistency of responses to subscale questions, measured by Cronbach’s alpha statistic, with ideal values ranging from 0.7 to 0.9^23^. Validity was assessed by examining the relationship of VF and QOL to PBDVA. Spearman correlation coefficients were used to quantify the association between visual acuity and the domains and subscales scores, and the Kruskal–Wallis test to compare scores across PBDVA categories. Mann–Whitney test was used to compare subscales scores between individuals enrolled and non-enrolled in visual rehabilitation programs from institutions for visually impaired. The association of overall health status, age, sex, and education with VF and QOL scores was investigated by multiple linear regression analysis.

For analysis, PBDVA were classified according to WHO distance vision impairment (VI) categories (no VI ≥ 20/40; mild VI < 20/40–20/63; moderate VI < 20/63–20/200; severe VI < 20/200–20/400; blindness < 20/400)^1^. Stata Statistical Software Release 14 (StataCorp LP, College Station, TX) was used for analysis with a significance level of *p* ≤ 0.05.

#### Rasch analysis

Winsteps® Rasch Analysis and Rasch Measurement software for persons & items was used to analyze the psychometric properties of the WHO-VFQ-20 with measures (in logits) of negative values representing better ability for person or higher difficulty for item. The overall performance of the instrument was assessed by Rasch-derived person and item separation indices. A minimum acceptable separation index for persons and items was set at 2.0 (reliability coefficient of 0.8), indicating the instrument’s capacity to differentiate at least two item difficulty and person ability levels^24–26^.

Item fit statistics, determined by infit (inliers fit) and outfit (outliers fit) values, were reported as a mean square (MNSQ) with an expected value of 1.0 (i.e. observed scores match the expected scores), given that the acceptable range considered for residual statistics (normalized to the expected mean variance) was 0.70 to 1.30^25^. To complement fit statistics, the unidimensionality (single latent trait) for items was assessed by comparing the raw variance explained by measures and the raw unexplained variance (in first contrast), both measured in eigenvalue units. Unidimensionality was established for empiric (expected) and modeled (observed) variance explained by the measures greater than 60%, indicating a low possibility of finding additional components^27^. Unexplained variance by the first contrast was also analyzed and multidimensionality was considered as eigenvalue > 2.0 (contrast greater than two items) which indicates that in the first contrast, the residuals (discrepancies between observed and expected data) of two items form a structure together, which may an additional dimension^25,27^.

## Results

This study included 606 participants (54.46% females) with ages ranging from 25 to 92 years (mean = 53.82 ± 17.00 s.d.). Table 1 shows the demographics and visual acuity distribution of the studied sample. Table 2 presents data on the self-reported general health status and the frequency of ophthalmological diagnoses. Of the 606 participants, 47.69% (n = 289) wore corrective spectacles, given that 54 (8.92%) were for only near vision, 77 (12.70%) only for distance vision, and 158 (26.07%) for both.

**Table 1:** Examined sample by age, sex, marital status, education level, monthly household income, and its distribution into presenting binocular distance visual acuity categories.

	Presenting binocular distance visual acuity categories											
	≥ *20/40* *No VI*		< *20/40–20/63* *Mild VI*		< *20/63–20/200* *Moderate VI*		< *20/200–20/400* *Severe VI*		< *20/400* *Blindness*		*Total*	
	*N*	*%*	*N*	*%*	*N*	*%*	*N*	*%*	*N*	*%*	*N*	*%*
*Age (yrs.)*												
25–39	55	36.91	7	4.70	35	23.49	14	9.40	38	25.50	149	24.59
40–49	38	40.86	3	3.23	22	23.66	8	8.60	22	23.66	93	15.35
50–59	44	39.64	7	6.31	20	18.02	13	11.71	27	24.32	111	18.32
60–69	45	32.61	15	10.87	46	33.33	12	8.70	20	14.49	138	22.77
> 70	30	26.09	14	12.17	38	33.04	18	15.65	15	13.04	115	18.98
*Sex*												
Male	97	35.14	15	5.43	63	22.83	34	12.32	67	24.28	276	45.54
Female	115	34.85	31	9.39	98	29.70	31	9.39	55	16.67	330	54.46
*Marital status*												
Single/lives alone	77	25.41	26	8.58	89	29.37	34	11.22	77	25.41	303	50.00
Married/cohabitating	135	44.55	20	6.60	72	23.76	31	10.23	45	14.85	303	50.00
*Education level*												
Less than primary	30	23.81	11	8.73	43	34.13	18	14.29	24	19.05	126	20.79
Primary	37	25.52	11	7.59	44	30.34	21	14.48	32	22.07	145	23.93
Secondary	43	39.45	12	11.01	23	21.10	13	11.93	18	16.51	109	17.99
High school	52	37.41	6	4.32	37	26.62	8	5.76	36	25.90	139	22.94
College/university	50	57.47	6	6.90	14	16.09	5	5.75	12	13.79	87	14.36
*Monthly household income (MW)*												
≤ 1	10	17.86	6	10.71	23	41.07	7	12.50	10	17.86	56	9.24
> 1–2	20	19.61	9	8.82	35	34.31	13	12.75	25	24.51	102	16.83
> 2–4	54	33.13	12	7.36	40	24.54	19	11.66	38	23.31	163	26.90
> 4–8	34	29.06	6	5.13	33	28.21	17	14.53	27	23.08	117	19.31
> 8–16	46	54.12	4	4.71	16	18.82	8	9.41	11	12.94	85	14.03
> 16	26	76.47	2	5.88	2	5.88	1	2.94	3	8.82	34	5.61
Not provided	22	44.90	7	14.29	12	24.49	0	0.00	8	16.33	49	8.09
Total	212	34.98	46	7.59	161	26.57	65	10.73	122	20.13	606	100.00

*MW* minimum wages, *VI* vision impairment.

**Table 2:** Frequency of ophthalmological diagnoses by presenting binocular distance visual acuity categories.

	**Presenting binocular distance visual acuity categories**											
	≥ *20/40*		< *20/40–20/63*		< *20/63–20/200*		< *20/200–20/400*		< *20/400*		*Total*	
	*N*	*%*	*N*	*%*	*N*	*%*	*N*	*%*	*N*	*%*	*N*	*%*
*Ophthalmological diagnoses*												
Normal	66	100.00	0	0	0	0	0	0	0	0	66	10.89
Unilateral ocular disease	44	97.78	1	2.22	0	0	0	0	0	0	45	7.43
Glaucoma	39	32.23	14	11.57	23	19.01	6	4.96	39	32.23	121	19.97
Other retinal disorder^a^	15	13.76	3	2.75	42	38.53	18	16.51	31	28.44	109	17.99
Diabetic retinopathy	16	14.68	13	11.93	48	44.04	15	13.76	17	15.60	109	17.99
Cataract	16	26.23	9	14.75	21	34.43	5	8.20	10	16.39	61	10.07
Corneal disorder^b^	9	45.00	5	25.00	4	20.00	1	5.00	1	5.00	20	3.30
Age-related macular degeneration	1	4.35	0	0	7	30.43	14	60.87	1	4.35	23	3.80
Other optic nerve disorder^c^	0	0	0	0	4	18.18	3	13.64	15	68.18	22	3.63
Uncorrected refractive error	0	0	0	0	4	80.00	1	20.00	0	0	5	0.83
Other^d^	6	24.00	1	4.00	8	32.00	2	8.00	8	32.00	25	4.13
Total	212	34.98	46	7.59	161	26.57	65	10.73	122	20.13	606	100.00

^a^ Includes: retinal dystrophies, maculopathy, high myopia, oculocutaneous albinism, phthisis bulbi, retinal detachment, retinal/vitreous hemorrhage.

^b^ Includes: bullous keratopathy, corneal ulcer, corneal transplant, pellucid marginal degeneration, Fuchs’ dystrophy, herpetic keratitis, keratoconus, measles-related corneal injury, pterygium.

^c^ Includes: compressive optic neuropathy, Leber’s hereditary optic neuropathy, optic atrophy, optic disc drusen, optic disc pallor, optic neuritis.

^d^ Includes: bulbar atrophy, coloboma, strabismus, uveitis.

Mean VF and QOL Likert scores are shown in Table 3. Out of all participants, 178 were enrolled in visual rehabilitation programs of the Fundação Dorina Nowill para Cegos and the Lar das Moças Cegas. Their WHO-VFQ-20 data were separately analyzed and are presented in Table 4.

**Table 3:** Visual function and quality of life mean Likert scores and standard deviations by presenting binocular distance visual acuity categories.

	**Presenting binocular distance visual acuity categories**												*p*
	≥ *20/40*		< *20/40–20/63*		< *20/63–200*		< *20/200–20/400*		< *20/400*		*Total*		
*N*	212		46		161		65		122		606		
	*mean*	*s.d*	*mean*	*s.d*	*mean*	*s.d*	*mean*	*s.d*	*mean*	*s.d*	*mean*	*s.d*	
General vision	60.26	(25.14)	45.65	(19.22)	40.22	(20.57)	30.77	(19.15)	14.96	(23.10)	41.54	(28.01)	< 0.001
Visual functioning	81.25	(19.27)	68.57	22.17	53.71	(19.30)	41.83	(17.77)	21.70	(16.16)	56.76	(29.07)	< 0.001
Distance vision	81.31	(23.01)	64.40	(25.71)	50.43	(21.88)	39.71	(18.86)	23.36	(17.55)	55.69	(30.67)	< 0.001
Near vision	84.35	(19.18)	70.79	(23.72)	57.38	(23.54)	40.19	(21.11)	18.24	(18.12)	58.11	(32.22)	< 0.001
Sensory adaptation	78.10	(21.29)	70.52	(22.54)	53.34	(21.48)	45.58	(22.06)	23.51	(19.80)	56.47	(29.34)	< 0.001
Quality of life	77.71	(20.22)	68.17	(23.02)	56.97	(21.83)	51.92	(20.88)	56.50	(21.91)	64.44	(23.61)	< 0.001
Ocular pain	76.77	(23.72)	72.28	(23.11)	72.20	(27.10)	77.31	(26.78)	71.52	(29.69)	74.22	(26.24)	0.415
Social interaction	84.51	(23.17)	72.10	(28.88)	57.57	(25.77)	46.67	(23.84)	45.49	(24.46)	64.49	(29.48)	< 0.001
Mental well-being	71.66	(23.78)	61.96	(25.86)	51.14	(26.88)	48.72	(28.95)	62.30	(28.79)	61.12	(27.84)	< 0.001
Vision-related quality of life	80.01	(18.70)	68.28	(21.56)	54.89	(18.10)	45.55	(16.53)	34.49	(15.02)	59.59	(24.96)	< 0.001

**Table 4:** Visual function and quality of life mean Likert scores and standard deviations by presenting binocular distance visual acuity categories for individuals enrolled in visual rehabilitation programs evaluated in the institutions for visually impaired (Fundação Dorina Nowill para Cegos and Lar das Moças Cegas).

	**Presenting binocular distance visual acuity categories**												*p*
	≥ *20/40*		< *20/40–20/63*		< *20/63–200*		< *20/200–20/400*		< *20/400*		*Total*		
*N*	3^a^		2^b^		48		39		86		178		
	*mean*	*s.d*	*mean*	*s.d*	*mean*	*s.d*	*mean*	*s.d*	*mean*	*s.d*	*mean*	*s.d*	
General vision	25.00	(43.30)	75.00	(35.36)	43.75	(19.64)	31.41	(18.78)	12.79	(23.87)	26.12	(26.15)	< 0.001
Visual functioning	34.03	(19.36)	75.00	(23.57)	48.83	(17.67)	36.43	(15.54)	19.50	(13.85)	31.99	(20.30)	< 0.001
Distance vision	27.08	(26.02)	75.00	(35.36)	43.88	(17.56)	36.38	(16.84)	21.22	(14.55)	31.36	(19.47)	< 0.001
Near vision	43.75	(18.75)	84.38	(22.10)	55.21	(24.41)	34.29	(17.20)	16.93	(17.07)	32.27	(25.67)	< 0.001
Sensory adaptation	31.25	(16.54)	65.63	(13.26)	47.39	(19.96)	38.62	(21.41)	20.35	(17.91)	32.34	(22.69)	< 0.001
Quality of life	55.95	(16.10)	71.42	(20.20)	60.12	(24.18)	52.75	(21.81)	60.90	(19.37)	58.89	(21.33)	0.282
Ocular pain	91.67	(14.43)	87.50	(17.68)	77.60	(26.42)	75.00	(26.28)	73.84	(29.43)	75.56	(27.61)	0.830
Social interaction	47.22	(12.73)	79.17	(29.47)	59.42	(26.09)	46.37	(25.92)	48.84	(21.93)	51.54	(24.48)	0.042
Mental well-being	52.78	(26.79)	58.33	(11.79)	54.69	(30.89)	51.71	(30.18)	68.41	(26.60)	60.67	(29.25)	0.020
Vision-related quality of life	42.10	(14.47)	73.68	(22.33)	52.99	(18.33)	42.44	(15.99)	34.72	(12.96)	41.90	(17.31)	< 0.001

^a^Individuals with visual acuity of 20/32 and constricted visual field (tunnel vision) due to diagnosis of retinitis pigmentosa.

^b^One individual with retinitis pigmentosa (20/50) and one individual with diabetic retinopathy (20/63).

The WHO-VFQ-20 internal consistency was excellent, with Cronbach’s alpha coefficient of 0.94 for the overall scale. The internal consistency of the VF and QOL domains were 0.94 and 0.78, respectively. The subscales Cronbach’s alpha coefficients were: 0.86 for distance vision; 0.85 for near vision; 0.83 for sensory adaptation; 0.73 for social interaction; 0.58 for mental well-being and dependency.

The WHO-VFQ-20 mean global score consistently reduced with decreasing visual acuity, as evidenced by Spearman correlation coefficients of 0.74 (*p* < 0.001). Significant correlations (*p* < 0.001) were found for near vision (r = 0.75), distance vision (r = 0.74), sensory adaptation (r = 0.71), social interaction (r = 0.60), and mental well-being (r = 0.24) subscales scores and PBDVA, except for the ocular pain (r = 0.04; *p* = 0.286) item. Comparing scores of individuals enrolled and non-enrolled in visual rehabilitation programs, those from the institutions for the visually impaired showed scores significantly lower (*p* < 0.001) for the subscales of distance vision, near vision, sensory adaptation, and social interaction, and comparable scores for the mental well-being (*p* = 0.922) subscale and the ocular pain (*p* = 0.221) item.

Higher VF was significantly associated with better overall health status (moderate: β = 8.19, *p* = 0.033; good/very good: β = 8.47, *p* = 0.030), higher education (High school/College: β = 12.08, *p* < 0.001) and being married/cohabitating (β = 11.14, *p* < 0.001). QOL score was significantly associated with better overall health status (moderate: β = 12.81, *p* < 0.001; good/very good: β = 21.50, *p* < 0.001), older age (≥ 60 years: β = 6.50, *p* = 0.010), higher education (Secondary/Primary: β = 8.16, *p* = 0.001; High school/College: β = 12.24, *p* < 0.001) and being married/cohabitating (β = 5.45, *p* = 0.003).

Table 5 presents the Rasch scores obtained for each item of the WHO-VFQ-20, which ranged from − 0.76 to 0.57 logits (mean = 0.00 ± 0.42 s.d.), all with a standard error of 0.04. Overall, the instrument demonstrated good precision with separation indices of 9.41 (reliability of 0.99) and 2.34 (reliability of 0.85) for item and person, respectively, indicating the differentiation of at least nine levels of item difficulty and two levels of person ability. For the VF domain, item difficulty (9.81) and person ability (2.36) results were similar to those found overall. QOL domain presented a person separation (1.34) lower than expected, but with acceptable item separation (9.86).

**Table 5:** Rasch and Likert scores of WHO-VFQ-20.

Item	*Question*	Ordinal Data Analysis		Rasch Analysis		
		Domain	Score mean (s.d.)	Score (logit)	Infit MNSQ	Outfit MNSQ
2	*How much pain or discomfort do you have in your eyes?*	QOL	74.22 (26.24)	0.57	1.26	1.99
18	*Because of your eyesight, how often have you found that you are ashamed or embarrassed?*	QOL	73.10 (35.36)	0.53	1.51	1.47
19	*Because of your eyesight, how often have your felt that you are a burden on others?*	QOL	72.57 (36.18)	0.51	1.23	1.05
7	*How much difficulty do you have in seeing differences in colors?*	VF	68.89 (38.35)	0.37	0.99	0.87
8	*Because of your eyesight, how much difficulty do you have in recognizing the face of a person standing near you?*	VF	67.99 (39.34)	0.33	1.06	0.87
16	*Because of your eyesight, how much difficulty do you have in carrying out your usual work?*	QOL	67.33 (31.80)	0.31	0.80	0.78
17	*Because of your eyesight, how often have you been hesitant to participate in social functions?*	QOL	66.71 (38.06)	0.28	1.29	1.16
3	*Because of your eyesight, how much difficulty do you have in going down steps or stairs?*	VF	65.80 (31.14)	0.25	0.79	0.82
9	*How much difficulty do you have in seeing the level in a container when pouring?*	VF	63.41 (38.55)	0.17	0.73	0.64
4	*How much difficulty do you have in noticing obstacles while you are walking alone?*	VF	63.20 (35.81)	0.16	0.68	0.61
6	*Because of your eyesight, how much difficulty do you have in searching for something on a crowed shelf?*	VF	62.75 (34.07)	0.14	0.75	0.71
10	*Because of your eyesight, how much difficulty do you have in going to activities outside of the house?*	QOL	59.28 (39.15)	0.02	0.73	0.72
13	*How much difficulty do you have seeing irregularities in the path when walking?*	VF	55.89 (38.56)	− 0.10	0.60	0.55
15	*How much difficulty do you have in doing activities that require you to see well close up?*	VF	51.07 (37.91)	− 0.27	1.11	1.06
12	*How much difficulty do you have in seeing close objects?*	VF	49.83 (39.63)	− 0.31	0.87	0.82
5	*How much difficulty do you have in seeing because of glare from bright lights?*	VF	49.26 (33.52)	− 0.33	0.85	0.89
14	*How much difficulty do you have in seeing when coming inside after being in bright sunlight?*	VF	44.88 (37.24)	− 0.49	0.83	0.82
1	*Overall, how would you rate your eyesight using both eyes?*	–	41.54 (28.01)	− 0.62	0.71	0.90
11	*Because of your eyesight, how much difficulty do you have in recognizing people you know from a distance of 20 m?*	VF	37.91 (40.29)	− 0.76	0.85	0.76
20	*Because of your eyesight, how often do you worry that you may lose your remaining eyesight?*	QOL	37.83 (41.33)	− 0.76	2.74	3.71

QOL: quality of life; VF: visual functioning.

The mean infit and outfit MNSQ statistics were, respectively, 1.02 ± 0.47 s.d. (range 0.60–2.74) and 1.07 ± 0.72 s.d. (range 0.64–3.71). Fit statistics were within the acceptable MNSQ range for most items, except for items 2, 4, 9, 13, 18, and 20 (Table 5), being around 0.6 for three of them (items 4, 9, and 13). The expected (51.6%) and observed (51.4%) variances explained by the measures were similar and acceptable but suggested the possible existence of an additional dimension. The variance explained by the measures was 21.12 eigenvalue units, and the unexplained variance was 4.32 units, indicating that the residuals in the first contrast of four items formed a structure together. Four items loaded positively (correlation > 0.4) onto the first contrast and were related to mental health (items 18, 20), social interaction (item 17), and pain (item 2). Seven items loaded negatively (correlation < − 0.4) onto the first contrast and were related to near vision (items 8, 9, 12), distance vision (items 4, 11, 13), and sensory adaptation (item 7). This suggests that these items should not be combined on the scale to measure a single latent trait, as they are related to two distinct components: visual functioning and psychosocial functioning.

Excluding misfitting items or those with high loadings in the dimensionality analysis did not improve the instrument’s fit to the Rasch model and thereby misfit other items. Therefore, new Rasch analyses were performed separately, considering two distinct components: visual functioning (items 3 to 15) and psychosocial functioning (items 1, 2, and 16 to 20).

**Fig. 2:**
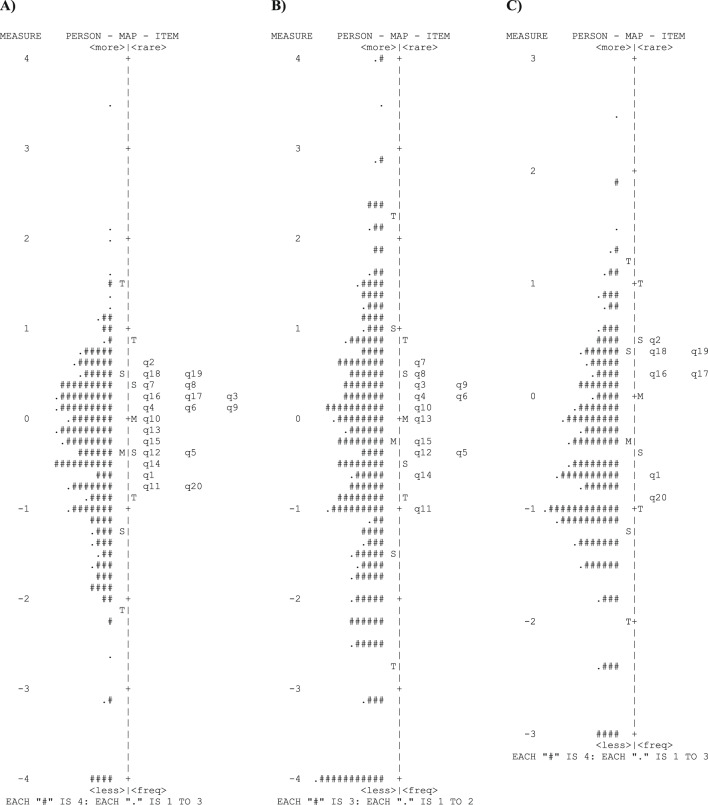
Wright item-person maps of the 20-item WHO Visual Functioning Questionnaire (WHO-VFQ-20) (**A**) and its components visual functioning (**B**) and psychosocial functioning (**C**). In each map, the left-hand column locates the person ability measures and the right-hand column locates the item difficulty measures along the scale. *M* mean, *S* one standard deviation from the mean, *T* two standard deviations ﻿from the mean, *q* question.

In this approach, the data showed improved model fit. Table 6 presents the results of precision, fit statistics, and dimensionality for both components. In visual functioning component, items 3 (infit and outfit MNSQ of 1.43 and 1.73, respectively) and 15 (infit and outfit MNSQ of 1.69 and 2.20, respectively) were misfitting, and loaded positively (correlation > 0.4) onto the first contrast along with item 6. In the psychosocial functioning component, only item 20 (infit and outfit MNSQ of 1.58 and 1.59, respectively) was misfitting. Figure 2 shows the Wright item-person maps of the two components of the WHO-VFQ-20.

**Table 6:** Rasch Analysis fit statistics of the visual functioning and psychosocial functioning components from the WHO-VFQ-20.

	Statistics	Component	
		Visual functioning	Psychosocial functioning
Measure	Mean (s.d.) score (logit)	0.00 (0.46)	0.00 (0.51)
Person	Separation	2.43	1.51
	Reliability	0.85	0.70
Item	Separation	9.48	12.20
	Reliability	0.99	0.99
	Misfitting items (n)	2 (3 and 15)	1 (20)
Variance	Expected	62.4%	51.0%
	Observed	61.6%	51.5%
	Raw variance explained by measures (eigenvalues)	20.82	7.29
	Raw unexplained variance in the first contrast (eigenvalues)	2.58	1.66

## Discussion

In this study, the WHO-VFQ-20 was applied in a larger and heterogeneous group of participants stratified by age, sex, educational level, socioeconomic status, and visual acuity level. This instrument was field-tested as recommended by WHO consultants for blindness prevention to validate its use for diverse populations from developing countries, since it was designed for them^22^. Data collection was conducted in the interviewer-administered format to ensure a reliable collection of data^28,29^, reducing the likelihood of missing data and doubts for individuals with visual, comprehension, or reading difficulties.

The current results demonstrated that the WHO-VFQ-20 Portuguese version is, in general, a reliable and valid instrument for measuring vision-related quality of life, with some caveats. Similar to the widely used NEI-VFQ-25^30–35^, overall scale Cronbach’s alpha of 0.9 showed that the instrument has good internal consistency^23^, a reliability indicator^36^. The WHO-VFQ-20 mean global score in our sample reduced significantly with decreasing visual acuity, as did the subscale scores (mainly distance vision, near vision, and sensory adaptation), except for the ocular pain item^37^, as previously reported^12,37–40^. Better overall health status, higher education, and being married/cohabitating were significantly associated with better visual functioning and quality of life. Additionally, older age was significantly associated with better quality of life. Previous work has suggested that higher education may lead to lower levels of emotional and physical distress, which may influence responses to the quality-of-life questionnaire^41^. Similarly, the ageing process may also contribute to minimizing emotional aspects and improving the quality of life in stressful situations related to physical health.

In addition to demographic factors, other potential mediators can affect quality of life independently of visual function, such as health literacy, which in turn is closely associated with social determinants^42^. Individuals with higher socioeconomic and educational levels have greater health literacy, since these factors influence their ability to seek, understand, and use information for self-care, health, and well-being, thus improving quality of life^43^. In this context, health literacy can facilitate access to eye care and help prevent visual function loss. Furthermore, lower WHO-VFQ-20 scores in participants with less schooling might not necessarily reflect negative impact from vision impairment on quality of life, but may be linked to lack of comprehension and misinterpretation of the questions content due to low health literacy. In the current study we believe that this aspect was minimized since the questionnaire was administered by personal interview.

It is important to emphasize that a high reliability index does not inherently indicate that the instrument is valid, that it is able to accurately measure what it intends to measure (a precision indicator). Cronbach’s alpha requires some assumptions, including sample heterogeneity, instrument validity, and unidimensionality^36^.

On the other hand, ordinal data analysis of items responses in the Likert scale also assumes a single latent trait measured by the instrument and ordinal response categories presume equidistant intervals among them^40,44^. Thus, in case of a multidimensional instrument, the use of raw scores can hide important differences between dimensions, and the mean score can also mask opposing behavior across dimensions^15^. Another caution is the ceiling or floor effects, which occur when the instrument is unable to discriminate between individuals with high (high ability) or low (extreme disability) levels of ability/function^15,44^. This can also occur with skewness items whose response categories are poorly discriminative at the ends of the scale, leading to reduced variability, masking true differences and resulting in an underestimation of high abilities or an overestimation of low abilities in the calculated global score^15,44^. In our sample, these factors may complicate the interpretation of the global score and discrimination of individuals with varying levels of vision-related quality of life, even when they have similar visual acuity, particularly those with severe visual impairment or exceptional visual function.

Therefore, in the current study, the WHO-VFQ-20 validity and its psychometric properties were highlighted by Rasch analysis. The WHO-VFQ-20 overall showed good precision with separation indices above 2.0^25,26^ for both item and person, indicating the instrument’s capacity to differentiate levels of item difficulty and person ability. Reliability derived from the separation index is a statistic comparable to Cronbach’s alpha, with higher values indicating greater reliability^45,46^. Our findings align with a study on the psychometric properties of the NEI-VFQ-25 in Brazilian Portuguese^40^ and other validity studies of the NEI-VFQ-25 including patients with different ocular diseases^37,38,44–47^.

Regarding fit statistics, 14 items were within the acceptable range of 0.7 to 1.3^25^, and other three items (4, 9, 13) were close to this range, with fit statistics of 0.6. The literature points out that for clinical observations, the range of 0.5 to 1.5 for the MNSQ statistics can be interpreted as a productive result for measurement^27,48,49^. The highest discrepancies were observed in items related to ocular pain (item 2) and mental well-being (items 18 and 20), which had more extreme scores on the scale, given that the misfit of these items was also reported by other researchers in the Rasch analysis of the NEI-VFQ-25^37,38,40,44,46,47^. This suggests that these items, which encompass ocular/visual symptoms and socioemotional aspects, likely showed limited alignment with the visual functioning in daily life. Considering the mental well-being subscale, collecting emotional data through interviews also may introduce response biases due to discomfort, shame, and social acceptance^28,29,31,33,34,37^. For the pain item, interestingly, several NEI-VFQ-25 studies in the literature, despite not using Rach analysis, reported low internal consistency and reliability^30,32–35,50^. This finding suggests a low impact of ocular pain on vision-related quality of life, supported by our ordinal data analysis results, which revealed that scores for the ocular pain item were not correlated with visual acuity and participation in visual rehabilitation programs.

The dimensionality analysis of the WHO-VFQ-20 revealed the possible existence of two dimensions, as previously reported in the literature for the NEI-VFQ-25^37,38,40,44,51^. Additionally, four items (2, 17, 18, 20) loaded positively, while seven items (4, 7–9, 11–13) loaded negatively onto the first contrast, thereby establishing the psychosocial and visual functioning components, respectively. The existence of an additional dimension has been previously reported, based on principal component analysis in the NEI-VFQ-25^37,38,44^. Two components—visual functioning (including distance and near vision items) and socioemotional (encompassing mental health and dependency items)—had also been identified in a Brazilian study^40^.

To improve the overall instrument fit to the model, one reported alternative is to remove misfit items or cluster response categories^52^, but these strategies may lead to negative consequences, including increased data variance and misfits of other items^25,52^. However, in the present study, removing the most misfitting items or items with high loadings on the dimensionality analysis did not improve the instrument’s fit to the Rasch model, since it misfitted other items. This can also occur when questions not applicable to specific populations are omitted, as in the NEI-VFQ-25 application in rural areas. Our alternative was to perform Rasch analyses treating the two components (visual functioning and psychosocial functioning) as separate instruments, which yielded improved model fit.

The assessment of the overall impact of ocular diseases and treatments, enabled by vision-related quality of life questionnaires^29^, combined with intervention results, can contribute to public policies geared toward disease prevention, healthcare, and promotion^53^. Therefore, the analysis derived from vision-related quality-of-life questionnaires requires care and thoroughness. And when choosing a research instrument, it is crucial to consider the study population’s characteristics and context^25,51^, as well as the need for cultural adaptation in translated versions^54^. This includes ensuring the instrument remains up to date. Considering WHO-VFQ-20, the examples of daily activities listed in some items can be adapted to the cultural/current context, while retaining the central content question. For instance, item 12 could include “reading messages on a smartphone” for today’s urban population.

Our findings demonstrated that the Portuguese version of the WHO-VFQ-20 is psychometrically acceptable mainly when analyzed in two components (visual functioning and psychosocial functioning) for assessing vision-related quality of life in adults with diverse clinical and demographic characteristics. This brief instrument may be useful in vision research, offering the advantage of being culturally more suitable for the middle to low-income populations and shorter than the NEI-VFQ-25. Although our findings are similar to those of studies^37,38,40,44,46,47^ using the NEI-VFQ-25, a direct scores comparison is not possible because the instruments differ. Moreover, future technological advancements in eye care may enable early diagnosis and treatment, thus improving vision-related quality of life and WHO-VFQ-20 scores.

Further studies in diverse samples, with different sociodemographic profiles, specific ocular diseases or visual conditions, are necessary to investigate and confirm the psychometric properties of this instrument in measuring the impact of ocular diseases and the outcomes of interventions, contributing to clarifying the frequently observed discrepancies between clinical measures and patient perspectives. For both clinical and research use, it is recommended that the WHO-VFQ-20 be analyzed into the two distinct components, related to visual and psychosocial functioning.

## Data Availability

The datasets used and/or analyzed during the current study are available from the corresponding author on reasonable request.
